# World Health Organization’s Growth Reference Overestimates the Prevalence of Severe Malnutrition in Children with Sickle Cell Anemia in Africa

**DOI:** 10.3390/jcm9010119

**Published:** 2020-01-02

**Authors:** Djamila L. Ghafuri, Shehu U. Abdullahi, Binta W. Jibir, Safiya Gambo, Halima Bello-Manga, Lawal Haliru, Khadija Bulama, Fahd M. Usman, Awwal Gambo, Muktar H. Aliyu, Brittany C. Greene, Adetola A. Kassim, Chris Slaughter, Mark Rodeghier, Michael R. DeBaun

**Affiliations:** 1 Vanderbilt-Meharry Sickle Cell Center for Excellence, Department of Pediatrics, Vanderbilt University School of Medicine, Nashville, TN 37203, USA; Djamila.labib@vumc.org (D.L.G.); Brittany.covert@vumc.org (B.C.G.); 2 Department of Pediatrics, Bayero University/Aminu Kano Teaching Hospital, 700233 Kano, Nigeria; Dr_suak@yahoo.com; 3 Department of Pediatrics, Murtala Mohammed Specialist Hospital, 700251 Kano, Nigeria; Bintajibir@gmail.com (B.W.J.); Saphiaaa01@gmail.com (S.G.); 4 Department of Hematology and Blood Transfusion, Barau Dikko Teaching Hospital/Kaduna State University, 800241 Kaduna, Nigeria; Mamanzara@yahoo.co.uk; 5 Department of Pediatrics, Barau Dikko Teaching Hospital/Kaduna State University, 800241 Kaduna, Nigeria; Drdaddy002@yahoo.com; 6 Department of Administration, Aminu Kano Teaching Hospital, 700233 Kano, Nigeria; Khadijabulama@yahoo.com (K.B.); ufahd@yahoo.com (F.M.U.); 7 Department of Administration, Murtala Muhammad Specialist Hospital, 700251 Kano, Nigeria; Awwalgambo@gmail.com; 8 Department of Health Policy, Vanderbilt Institute for Global Health, Vanderbilt University School of Medicine, Vanderbilt University Medical Center, Nashville, TN 37203, USA; Muktar.aliyu@vumc.org; 9 Department of Hematology and Oncology, Vanderbilt University School of Medicine, Vanderbilt University Medical Center, Nashville, TN 37232, USA; Adetola.kassim@vumc.org; 10 Department of Biostatistics, Vanderbilt University School of Medicine, Nashville, TN 37203, USA; james.c.slaughter@vumc.org; 11 Rodeghier Consultants Chicago, Chicago, IL 60631, USA; Markrodeghier@comcast.net

**Keywords:** sickle cell disease, growth references, severe malnutrition

## Abstract

Anthropometric indices are widely used to assess the health and nutritional status of children. We tested the hypothesis that the 2007 World Health Organization (WHO) reference for assessment of malnutrition in children with sickle cell anemia (SCA) overestimates the prevalence of severe malnutrition when compared to a previously constructed SCA-specific reference. We applied the WHO and SCA-specific references to children with SCA aged 5–12 years living in northern Nigeria (Primary Prevention of Stroke in Children with SCA in sub-Saharan Africa (SPRING) trial) to determine the difference in prevalence of severe malnutrition defined as body mass index (BMI) *Z*-score <−3 and whether severe malnutrition was associated with lower mean hemoglobin levels or abnormal transcranial Doppler measurements (>200 cm/s). A total of 799 children were included in the final analysis (median age 8.2 years (interquartile range (IQR) 6.4–10.4)). The application of the WHO reference resulted in lower mean BMI than the SCA-specific reference (−2.3 versus −1.2; *p* < 0.001, respectively). The use of the WHO reference when compared to the SCA-specific reference population also resulted in a higher prevalence of severe malnutrition (28.6% vs. 6.4%; *p* < 0.001). The WHO reference significantly overestimates the prevalence of severe malnutrition in children with SCA when compared to an SCA-specific reference. Regardless of the reference population, severe malnutrition was not associated with lower mean hemoglobin levels or abnormal transcranial Doppler (TCD) measurements.

## 1. Introduction

Sickle cell anemia (SCA) is one of the most common monogenic disorders [1] and disproportionately impacts countries in sub-Saharan Africa, like Nigeria. Of the 305,000 annual births of children with SCA worldwide [2], about 150,000 births occur in Nigeria [3] as compared to approximately 1300 births in the United States [4]. The effects of sickle cell disease (SCD) on nutritional status were first described in the 1950s and 1960s [5,6]. Children with SCD, similar to children with other chronic diseases, are at risk for delayed growth and pubertal maturity [7,8,9]. The largest prospective study of more than 2000 children and young adults with various sickle genotypes describing the natural history of SCD (Cooperative Study of Sickle Cell Disease (CSSCD)) demonstrated that individuals with HbSS and HbSβ0 thalassemia (referred to here as SCA) had lower weights and shorter heights compared to those with HbSC and HbSβ+ thalassemia. Furthermore, all four SCD genotypes were below norms for African Americans [8]. A comprehensive review of 46 anthropometric studies (26 cross-sectional and 20 longitudinal) revealed similar findings of growth failure worldwide among individuals with SCD [9].

In children with a chronic disease, the most widely acceptable method of assessing nutritional status is to use a reference population in children with the same chronic disease [10,11,12,13,14,15,16]. However, most studies describing the growth patterns of children with SCD apply the World Health Organization (WHO) growth anthropometrics, which are based on normal growth in children without SCD. Often-cited disease-specific growth charts for chronic disease include Down syndrome [12,15,16], Turner syndrome [10,14], and cystic fibrosis [11,13]. Wolf et al. constructed reference percentiles for weight, height, and blood pressure measurements for male and female children with SCA between five and 15 years of age [17]. However, this study did not include the expected body mass index (BMI) percentiles, the standard metric for assessment of severe malnutrition [18]. The absence of SCA-specific growth curves for children with SCA, particularly in low-income settings, results in misclassification of malnutrition and misappropriation of limited resources required to treat malnutrition. However, until such information is available, WHO references for growth parameters will continue to be applied to children with SCA.

To address the gap in knowledge regarding the appropriate anthropometric standard in children with SCA, we tested the hypothesis that the WHO growth reference [18] in children with SCA overestimates the prevalence of severe malnutrition when compared to a growth reference specifically constructed using longitudinal anthropometric measurements of children with SCA living in high-income countries [17]. We also tested the hypothesis that severe malnutrition in children with SCA would be associated with lower mean hemoglobin levels or abnormal transcranial Doppler measurements (>200 cm/s).

## 2. Materials and Methods

### 2.1. Study Design and Population

We conducted a cross-sectional study of 850 children with SCA aged 5–12 years old living in the low-resource setting of northern Nigeria. We compared SCA-specific and WHO growth references to characterize the nutritional status of the population.

Children in this cross-sectional study were eligible participants for a National Institute of Health (NIH)-funded randomized controlled trial; the Primary Prevention of Stroke in Children with SCA in sub-Saharan Africa (SPRING Trial, NCT02560935), from July 2016 to July 2017. Participants were recruited from two states in northern Nigeria (estimated population in 2006: 2,828,861) providing medical care for an estimated 32,000 children with SCA [19]. 

Ethical approval for this study was obtained from all participating sites, and all participants provided informed consent: the coordinating center Vanderbilt University Medical Center in Nashville, Tennessee, United States of America (USA), and the local clinical sites Aminu Kano Teaching Hospital and Murtala Mohammad Specialist Hospital, with referrals from Hasiya Bayero Pediatric Hospital and Muahmmad Abdullahi Wase Specialist Hospital, all in in Kano, Nigeria and Hospital, Barau Dikko Teaching in Kaduna, Nigeria.

○ Aminu Kano Teaching Hospital (AKTH) is a 500-bed tertiary level facility. The pediatric SCD clinic at AKTH has a total patient pool of about 2010, with approximately 80 children with SCA seen weekly, staffed by a pediatrician and a nurse;
○ Murtala Muhammad Specialist Hospital (MMSH) is an AKTH-affiliated high-volume clinic located less than three miles from AKTH. The pediatric clinic runs daily (Monday through Friday) and has a total patient pool of over 17,810 children with SCA, with at least 400 children seen each week;
○ Hasiya Bayero Pediatric Hospital (HBPH) is a 90-bed pediatric hospital located approximately 2.5 miles from AKTH and is the only hospital in the area dedicated to the care of children. HBPH runs a full pediatric SCD clinic once a week with a total patient pool of over 11,129 children with SCA. Nurses and community health workers evaluate over 100 children with SCA daily and pediatricians once a week;
○ Muhammad Abdullahi Wase Specialist Hospital (MAWSH) is a 320-bed multispecialty hospital and the third busiest hospital in Kano, after MMSH and AKTH. MAWSH is located five miles from AKTH with a total pool of 470 children with SCA. Approximately 42 children with SCA are seen once a week by a pediatrician and a nurse;
○ Barau Dikko Teaching Hospital (BDTH) serves as the teaching hospital for Kaduna State University. The SCD Clinic is open once a week, with an estimated total 1200 children with SCD registered and an average of 40 children seen weekly.

### 2.2. SCA-Specific Growth Reference

The Silent Cerebral Infarct Multi-Center Clinical (SIT) trial is a randomized two-armed controlled clinical trial in which children between five and 15 years with SCA and silent cerebral infarct were allocated to three years of chronic transfusion therapy or observation (ClinicalTrials.gov: NCT00072761) [20]. The SIT trial recruited participants from four high-income countries, including the United States, Canada, France, and United Kingdom, between December 2004 and November 2013 [20]. Wolf et al. constructed reference percentiles for weight, height, and body mass index (BMI) for males and females with SCA, which are readily available for medical use [17]. Wolf et al. constructed the growth charts, using serial height and weight measurements from a total of 949 participants with a median age of 8.2 years (interquartile range (IQR) 6.4–10.4) and a median follow-up time of 3.2 years (IQR: 1.8–4.7, range 0–12.9). Participants had a median of five height and weight measurements (IQR: 3–7, range 1–12) [17]. Quantile regression was used to generate estimates of the percentiles of height and weight by age and sex [17].

### 2.3. 2007 WHO Growth Reference

The WHO 2007 growth reference data are intended to reflect optimal growth parameters and are based on high-quality studies of school-aged children and adolescents [18]. The 2007 WHO growth charts for school-aged children and adolescents (5–19 years old) were constructed using data from three merged datasets. The first and second datasets were from the Health Examination Survey (HES) Cycle II (6–11 years; *n* = 7417) and Cycle III (12–17 years; *n* = 7514), a longitudinal study conducted in the United States [18]. The third dataset was from the United States and was the longitudinal National Health and Nutrition Examination Survey (NHANES) Cycle I (birth to 74 years), from which only data from the 1–24 age range were used (*n* = 2878 children between five and 12 years of age). The final 2007 WHO growth reference sample used for fitting the expected BMI-for-age curves included 30,018 observations (15,103 boys, 14,915 girls) [18].

### 2.4. Data Collection and Definitions 

Demographics, socioeconomic status, baseline clinical and laboratory values, including baseline hemoglobin, white blood count (WBC), mean corpuscular volume (MCV), and baseline blood pressure (mmHg) were collected. Anthropometric measures (height (cm) and weight (kg)) were collected by a study administrator, nurse, or physician. Height and weight were used to calculate body mass index (BMI, kg/m^2^). Weight and height measurements were converted to *Z*-scores for weight-for-height (WHZ) based on SCD-specific [17] and WHO growth references [18]. BMI was expressed as *Z*-scores to assess the nutritional status. We defined wasting as a BMI *Z*-score <−2, moderate malnutrition as a BMI *Z*-score between −2 and −3, and severe malnutrition as a BMI *Z*-score <−3. Children with BMI-for-age between the 85th and 94th percentile (or BMIZ-score >2 and <3) were considered overweight and those ≥95th percentile were considered obese. Underweight children were defined as participants with BMI-for-age <5th percentile, whereas those between the fifth and 84th percentile were considered normal weight. 

### 2.5. Statistical Analysis

The demographics and anthropometric characteristics of all participants were summarized as means and standard deviations for continuous variables or as medians and interquartile ranges for variables not normally distributed, and as numbers and percentages for dichotomous variables. Prevalence was summarized by proportions. Data from children with SCA between five and 12 years of age and enrolled in the SPRING cohort were used to determine the prevalence of malnutrition (wasting, severe, moderate malnutrition, overweight, and obesity) based on the WHO 2007 growth reference at each age and the SCA-specific data. Comparisons were made using a paired sample *t*-test for continuous parameters (BMI *Z*-scores) and McNemar’s test for dichotomous parameters (prevalence of malnutrition). Continuous or ordinal measures between two groups were compared by ANOVA or the Mann–Whitney test, if the continuous data were not normally distributed. To assess the association of malnutrition with transcranial Doppler (TCD), controlling for age and other covariates, a quantile regression model predicting the median was used because of the non-normal distribution of TCD. A two-sided *p*-Value of 0.05 was considered significant. Data analysis was performed using SPSS 25.0 (IBM, Armonk, NY, USA). 

## 3. Results

### 3.1. Demographics 

**Figure 1:**
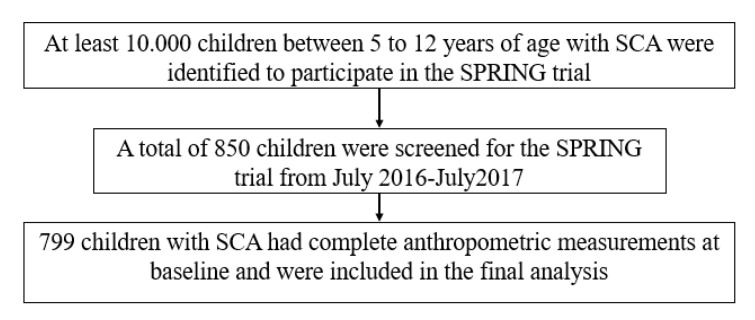
Participant recruitment flow diagram including children with sickle cell anemia screened for Primary Stroke Prevention in Nigeria (SPRING) trial.

Between July 2016 and July 2017, a total of 850 children with SCA were screened for the SPRING trial in Nigeria, of which 799 had complete anthropometric values at baseline and were included in the final analysis (Figure 1). The median age of children with SCA in the SPRING cohort was 8.2 years; approximately half were male (49.4%) (Table 1).

### 3.2. The Prevalence of Malnutrition Is Higher Based on the WHO Growth Reference Compared to SCD-Specific Growth Reference

The application of the WHO reference population resulted in lower mean BMI *Z*-scores when compared to the SCA-specific reference (−2.3 (2.0) and −1.2 (1.1); *p* < 0.001, respectively). Regardless of the reference population (WHO or SCA-specific reference), mean hemoglobin level and abnormal TCD measurements were not associated with severe malnutrition. The baseline characteristics and the nutritional status of the cohort are displayed in Table 1. The mean hemoglobin and proportion of abnormal TCD values categorized by nutritional status are displayed in Table 2 and Table 3, respectively. 

**Figure 2:**
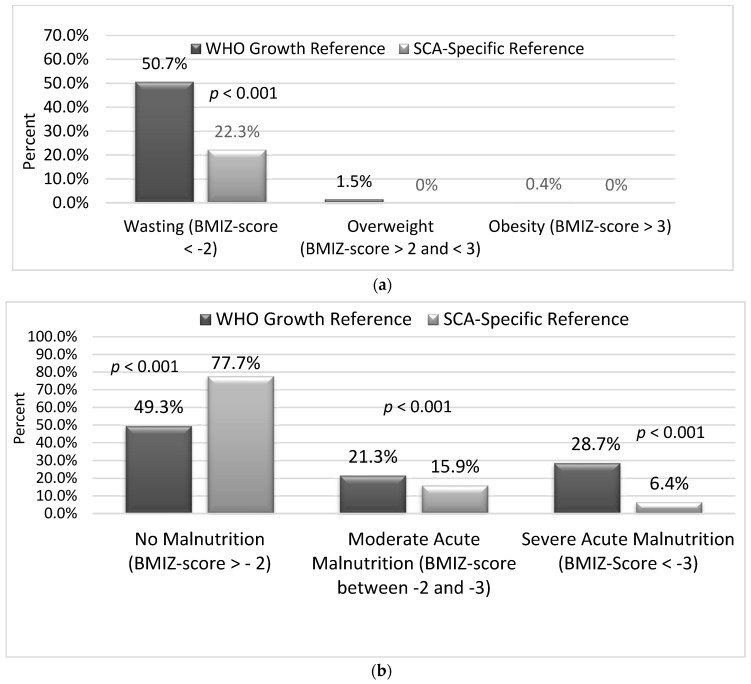
Proportion of children with sickle cell anemia (SCA) with malnutrition according to World Health Organization (WHO) and SCA-specific growth references. (**a**) Proportion of children with SCA defined as wasting, overweight, or obesity based on two reference populations. Using the WHO growth reference in children (5–12 years of age) with sickle cell anemia (SCA) living in northern Nigeria misclassified the prevalence of wasting when compared to the SCA-specific growth reference. McNemar’s test. A *p*-Value <0.05 was set for statistical significance. (**b**) Proportion of children with sickle cell anemia defined as having malnutrition. Using the WHO growth reference in children (5–12 years of age) with sickle cell anemia (SCA) living in northern Nigeria misclassified the prevalence of moderate malnutrition (body mass index (BMI) *Z*-score <−2 and >−3) and severe malnutrition (BMI *Z*-score <−3) in children with SCA living in Kano, Nigeria, categorized by WHO and SCA-specific growth reference. McNemar’s test. A *p*-Value  < 0.05 was set for statistical significance.

When we applied the WHO growth reference, the rate of wasting (BMI *Z*-score <−2) was significantly higher compared to the SCA-specific growth reference, 50.7% versus 22.3%, respectively (*p* < 0.001) (Figure 2a). The difference in prevalence of severe malnutrition was 22.2% higher with the WHO reference when compared to the SCA-specific growth reference (28.7% versus 6.4%, respectively, *p* < 0.001). The difference in prevalence of moderate malnutrition was 5.4% higher with the WHO reference when compared to the SCA-specific growth reference (21.3% versus 15.9%, respectively, *p* < 0.001) (Figure 2b). The prevalence of overweight and obesity based on the WHO reference versus the SCA-specific growth reference was 1.5% and 0.4%, respectively (Figure 2a).

**Figure 3:**
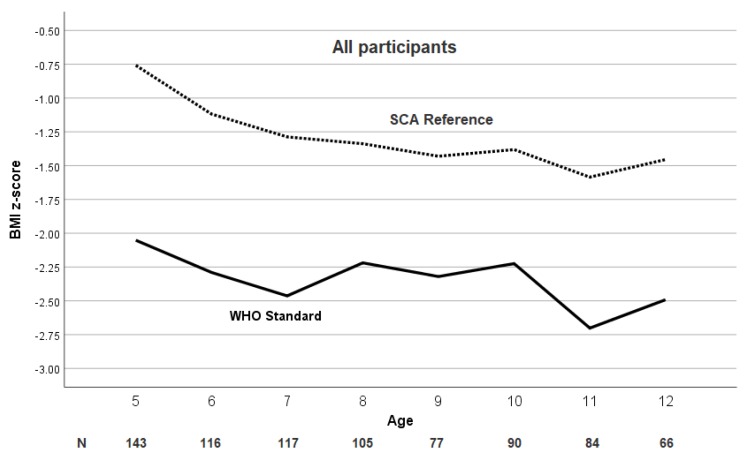
Age trend of BMI *Z*-score in children with SCA using the WHO and SCA reference populations. The mean BMI *Z*-score in children (5–12 years of age) with sickle cell anemia (SCA) in Nigeria is consistently greater based on the SCA-specific growth reference at each age compared to the WHO growth reference.

**Figure 4:**
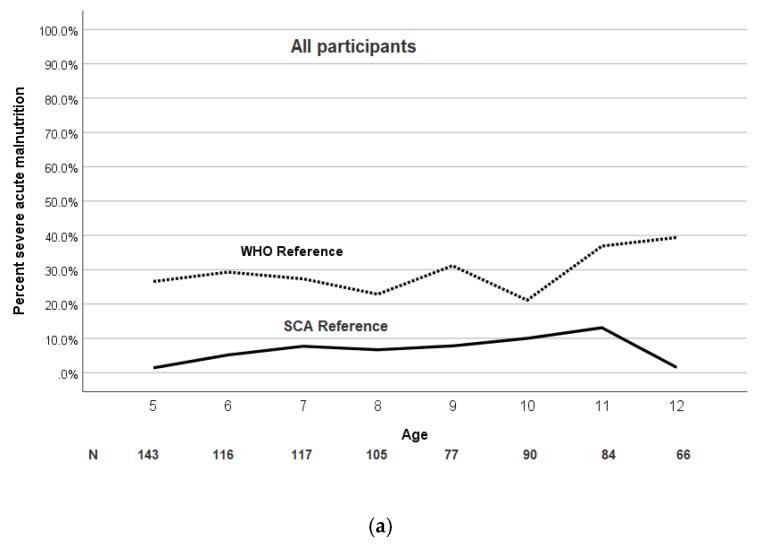

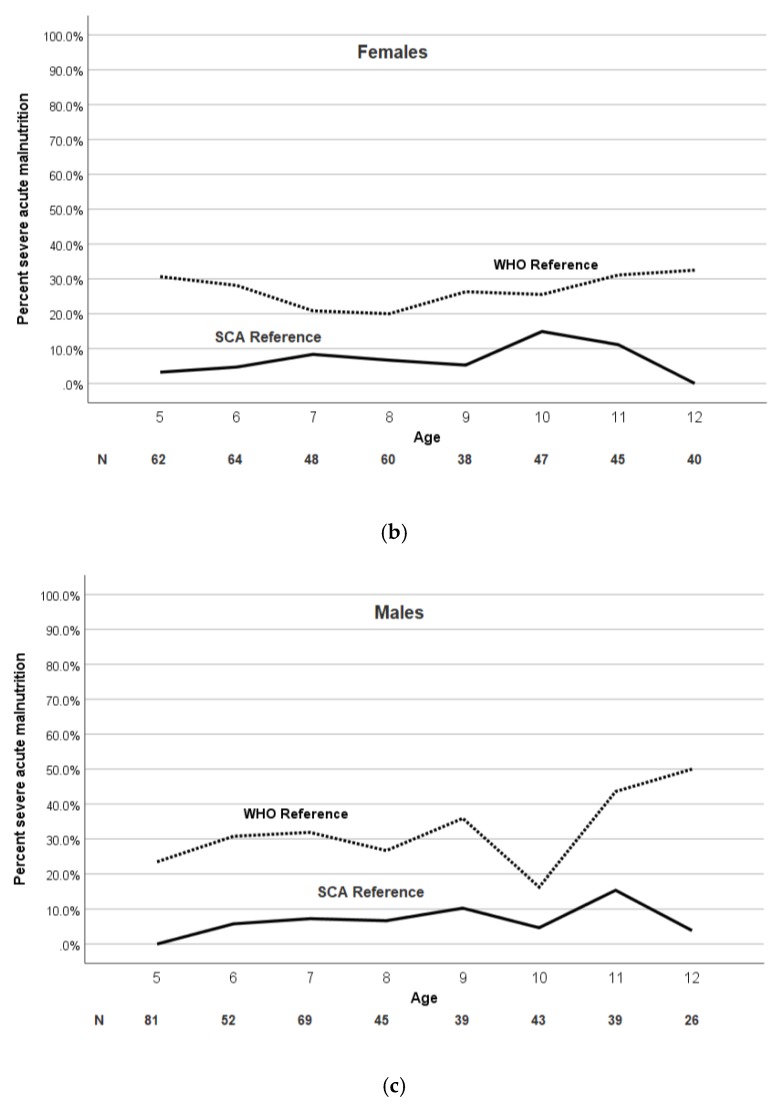
Age and sex trend of percentage severe malnutrition in children with SCA using the WHO and SCA reference populations. (**a**) The prevalence of severe malnutrition (BMI *Z*-score <−3) in children with SCA is dependent on the growth reference used and is consistently greater at each age according to the WHO growth reference. (**b**,**c**) Age and sex trend of BMI *Z*-score in children with SCA using the WHO and SCA reference population. The prevalence of severe malnutrition (BMI *Z*-score <−3) in both males and females with SCA is consistently greater at each age according to the WHO growth reference with an increase after age 10.

The mean BMI *Z*-score and prevalence of severe malnutrition was significantly and consistently higher at each age when using the WHO growth reference. The mean BMI *Z*-score and the prevalence of moderate and severe malnutrition at each age for the pediatric population are shown in Figure 3 and Figure 4a,b, respectively.

### 3.3. Children with Severe Malnutrition Have Lower Mean TCD Measurements Compared to Those without Malnutrition Based on the WHO and SCA-Specific Growth Reference

The unadjusted relationship between time-averaged maximum mean velocity (TAMMV) values and malnutrition category is significant. Children with severe malnutrition, according to both WHO and SCA-specific growth references, had lower mean TCD measurements (WHO: mean TCD 138.7 versus 150.2, respectively, *p* = 0.001; SCA-specific mean TCD 133.1 versus 147.6, respectively, *p* = 0.002) (Table 2). 

Based on the observation that TAMMV values decreased with age and the proportion of children with malnutrition increased with age, we performed a quantile regression model for TCD using the SCA-specific malnutrition categories (Appendix A, Table A1). Only moderate malnutrition is associated with a decline of −8.6 in TCD (*p* = 0.041), controlling for age and other relevant covariates. 

Regardless of the growth reference population, severe malnutrition was not associated with lower mean hemoglobin levels (Table 3). Severe malnutrition (BMI *Z*-score <−3) in children with SCA was not associated with increased prevalence of abnormal TCD measurements according to the WHO growth reference, when compared to children with SCA and no malnutrition (7.9% and 14.8%, respectively; *p* = 0.59). According to the SCA-specific growth reference, severe malnutrition in children with SCA was associated with lower prevalence of abnormal TCD measurements, when compared to children with no malnutrition (4.4% and 14%, respectively; *p* = 0.034).

## 4. Discussion

Given the magnitude of SCA coupled with the large number of children with severe malnutrition in sub-Saharan Africa (150,000 in Nigeria alone [3] when compared to the USA of approximately 1300 newborns with SCA per year [4], understanding the epidemiology and utilizing the appropriate growth reference to assess malnutrition is required to maximize appropriate distribution limited resources for this vulnerable population. To our knowledge, this is the first evidence to suggest that the WHO growth reference overestimates the proportion of children with severe malnutrition when compared to an SCA-specific reference in children aged 5–12 years.

In our cohort of older children with SCA, the prevalence of severe malnutrition was dependent on which growth reference was utilized. The prevalence of severe malnutrition in our study according to the SCA-specific growth parameters of children with SCA living in high-income setting was 6% vs. 28% based on the WHO reference. 

Given the evidence that malnutrition can be associated with low hemoglobin levels in the general population [21], we postulated that severe malnutrition in the SPRING cohort would be associated with abnormal TCD measurements. However, we found no evidence that severe malnutrition was associated with lower hemoglobin levels or abnormal TCD measurements, which was similar to the Jamaican study including 358 children with SCA [22]. The absence of an association between a low hemoglobin level and severe malnutrition might possibly be related to the observation that children with SCA are already anemic. In this study, enrolling children above five years of age possibly contributed to the lack of a relationship between Hb level and severe malnutrition, because severe malnutrition and anemia are more common in children below the age of five.

Even when only focusing on the narrow range of 5–12 years of age, a discordant pattern for classifying severe malnutrition was observed in the two growth references in our cohort. When using the WHO growth reference, an increase in the prevalence of severe malnutrition was noted at age five and at age 10 for both males and females (Figure 3a,b). The increase in prevalence of severe malnutrition at five years of age is possibly due to the relative lack of government or philanthropic support for the management of malnutrition for children over five years of age, when compared to the abundance of malnutrition programs in children less than five years of age. We postulate that the increase in prevalence of malnutrition at 10 years of age, when using the WHO reference, could be explained by the delay of puberty by approximately two years in children with SCA when compared to children without SCA [23]. 

Our study has several limitations. Due to the cross-sectional design, the clinical impact of poor nutritional status on overall health, morbidity, and mortality could not be assessed. The constructed SCA-specific growth reference from the SIT trial was based on secondary analyses; however, the SIT dataset included serial measurements with a median follow-up of 3.2 years (IQR 1.8–4.7; range 0–12.9 years) and represents the best data available from over 25 SCA clinics in four high-income countries. Another limitation is that the anthropometric data from an age-matched pediatric cohort without SCA in the same region of northern Nigeria was not selected. However, the appropriate comparison group to assess growth parameters is not children without SCA in a resource-limited setting with high rates of malnutrition, but children with SCA living in resource-limited settings where severe acute malnutrition is uncommon.

## 5. Conclusions

In a low-resource setting where malnutrition is endemic, accurate assessment of malnutrition in children with SCA is critical, because significant resources are required to treat and manage severe malnutrition. Our data provide compelling evidence that, for older children with SCD living in Nigeria, applying the WHO reference for defining severe malnutrition overestimates the proportion of children with severe malnutrition when compared to an SCA-specific reference of children living in high-income countries. 

## Figures and Tables

**Table 1:** Baseline characteristics of children with sickle cell anemia (SCA) aged 5–12 years old living in northern Nigeria and screened for the Primary Stroke Prevention in Nigeria (SPRING) trial (*n* = 799) compared to high-income countries (Silent Cerebral Infarct Multi-Center Clinical (SIT) cohort; *n* = 1127).

Variable	SPRING Cohort (*n* = 799)	SIT Cohort (*n* = 1127)	*p*-Value #
Age, median (IQR) (years)	8.2 (6.4–10.4)	8.5 (6.7–10.7)	0.001 §
Sex, male, *n* (%)	395 (49.4)	579 (51.4)	0.402
Weight, median (IQR)(kg)	19.0 (16.0–22.0)	25.9 (22.0–31.9)	<0.001 §
Height, median (IQR)(cm)	120.0 (111.0–128.0)	126.8 (117.9–137.7)	<0.001 §
BMI, mean (SD)	13.7 (1.9)	16.4 (2.7)	<0.001
Hemoglobin, mean (SD) (*n* = 1909)	7.6 (1.1)	8.2 (2.6)	<0.001
White Blood Count, mean (SD) (*n* = 1905)	14.7 (5.1)	12.6 (5.2)	<0.001

BMI, body mass index; Hb, hemoglobin; IQR, interquartile range; WBCs, white blood cells. # A *p*-Value < 0.05 was set for statistical significance. § Mann–Whitney U test.

**Table 2:** Proportion of children with sickle cell anemia (SCA) aged 5–12 years old in Nigeria (SPRING cohort; *n* = 799) with abnormal transcranial Doppler (TCD) values (>200 cm/s): World Health Organization (WHO) compared to the SCA-Specific growth reference.

Malnutrition	Abnormal TCD, %	*p*-Value #
SCA-Specific Growth Reference *n* = 799		
No malnutrition	14.0	0.034
Moderate malnutrition	7.1	
Severe malnutrition	4.4	
2007 WHO Growth Reference *n* = 799		
No malnutrition	14.8	0.059
Moderate malnutrition	12.3	
Severe malnutrition	7.9	

Abnormal TCD, >200 cm/s; moderate malnutrition, BMI *Z*-score <−2 and >−3; severe malnutrition, BMI *Z*-score <−3. # Chi-square. A *p*-Value  < 0.05 was set for statistical significance.

**Table 3:** Mean hemoglobin of children with sickle cell anemia (SCA) aged 5–12 years old in Nigeria (SPRING cohort; *n* = 799): WHO compared to the SCA-Specific growth reference.

Malnutrition	Hemoglobin (g/dL), Mean (SD)	*p-*Value #
SCA-Specific Growth Reference		
No malnutrition	7.5 (1.1)	0.124
Moderate malnutrition	7.6 (1.1)	
Severe malnutrition	7.9 (1.5)	
2007 WHO Growth Reference		
No malnutrition	7.5 (1.2)	0.981
Moderate malnutrition	7.6 (1.1)	
Severe malnutrition	7.6 (1.2)	

Moderate malnutrition: BMI *Z*-score <−2 and >−3; severe malnutrition, BMI *Z*-score <−3. # ANOVA. A *p*-Value < 0.05 was set for statistical significance.

## Appendix A

**Table A1:** Quantile regression model for transcranial doppler measurements in children with sickle cell anemia with SCA-specific malnutrition categories (*n* = 701). Moderate malnutrition is associated with a decline of −8.6 in TCD (*p* = 0.041), controlling for age and other relevant covariates.

Variable	Coefficient *	95% CI	*p*-Value
Age	−3.44	−4.80, −2.09	<0.001
Sex (male)	−3.27	−9.24, 2.69	0.282
Hemoglobin	−6.96	−10.04, −3.88	<0.001
White blood cell count	0.61	−0.06, 1.28	0.076
SCA-specific moderate malnutrition #	−8.57	−16.81, −0.34	0.041
SCA-specific severe malnutrition #	−10.44	−22.82, 1.94	0.098

* Coefficient predicting the median of TCD; CI, confidence interval. # Reference category is no malnutrition.
